# A General Pathway Model for Improving Health Disparities: Lessons from Community and Cultural Involvement in Improving Cervical Cancer Screening in Vietnamese Women

**DOI:** 10.3390/jcm8020154

**Published:** 2019-01-29

**Authors:** Richard Kones, Umme Rumana, Fauzia Arain

**Affiliations:** 1 Department of Cardiology, The Cardiometabolic Research Institute, Houston, TX 77054 USA; umme.rumana.mbbs@gmail.com; 2 New York Institute of Technology, Old Westbury, NY 11568, USA; 3 Alzheimer’s Disease Center, NYU Langone Medical Center, New York, NY 10016, USA; fauziaarain@yahoo.com

**Keywords:** Papanicolaou tests, pathway model, health-delivery models, community-based participatory research, health disparities, social determinants of health, community developed intervention, chronic disease model, lay health workers

## Abstract

Objective: Chronic diseases have become dominant in the global health landscape. Despite remarkable advances in basic science, pharmacology, surgery, and technology, progress in lifestyle improvements, now considered essential, has been disappointing. Patient adherence to medications and other instructions play the greatest role in individual outcome shortfalls. Classically medicine has approached management using a high-risk model, targeting clinical manifestations of disease with progressively intensive therapies, in contrast with population-based models. In an effort to identify effectiveness among the many models available, the “pathways model” is reevaluated. Methods: Relying upon secondary data from prior studies in which Papanicolaou (Pap) test utilization was successfully improved, a “pathway model” is qualitatively reexamined in which characteristics of patients, providers, and the health system—as impacted by culture, beliefs, values, and habits—are acknowledged and incorporated by community resources into treatment plans. In so doing, health disparities are also addressed. Observations: The culturally inclusive pathways model using immersion community-based participation was successful in modifying behaviors when applied to a high-risk population in great need of improving Pap test adherence. Conclusions: In populations characterized by recognized cultural barriers contributing to low adherence, the pathways model may improve chronic disease outcomes. This model emphasizes a high degree of immersion within a culture and community as vehicles to improve patient behavior and address inequities. Central features are concordant with current concepts in guidelines, scientific statements, manuals, and advisories concerning the conduct of community-based research and social determinants of health. The pathways model deserves consideration for use in other chronic illnesses, such as cardiometabolic disease.

## 1. Introduction

During the past decade, the proposal that wellness, well-being, and health disparities are interconnected, and that effective health care delivery must involve recognition of social determinants of health, including socioeconomic status, has gained considerable traction [1,2,3,4,5,6,7,8,9,10,11,12,13,14,15,16,17,18,19,20,21,22,23,24,25,26,27,28,29,30,31,32,33,34,35,36,37,38,39]. One current major public health challenge involves recruitment of all resources in order to optimize health care outcomes, with the corollary being: what works best, when, where, and in which subpopulation [20,21,22,23,24,25,26,27,28,29,30]? 

Culture is a broad term but includes common and collective customs, values, beliefs, habits, and behaviors, a simplified version of “the shared patterns of behaviors and interactions, cognitive constructs, and affective understanding that are learned through a process of socialization” which identify and distinguish members of a group [31]. Within this umbrella, there is room for an additional dimension of individual characteristics that also influence and refine these same variables—gender, personality, age, family, experience, education, socioeconomic and political role within a community, capability, philosophy, and scope of interactions [32]. Vietnamese women are the 4th largest subgroup of Asians, the fastest-growing, rapidly-assimilating minority in the US. Immigration of the Vietnamese accelerated after the fall of Saigon in the Vietnamese War (1975), who settled chiefly in California and Texas [33]. In the 1980s this was followed by “boat people” who were generally less healthy, less educated, and primarily composed of rural dwellers [34]. 

**Figure 1:**
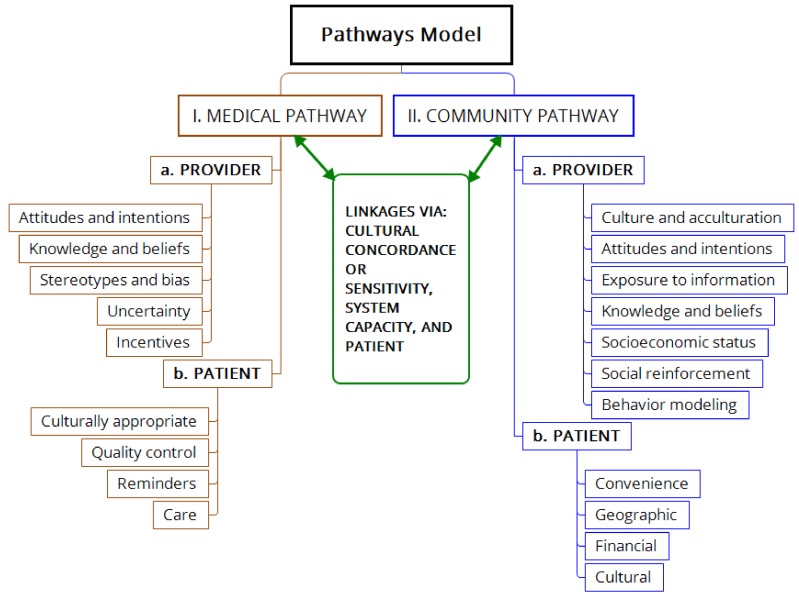
Conceptual diagram of the pathways model of health delivery which combines a medical pathway (left vertical) and a community pathway (right vertical), each of which involve both provider and patient characteristics. Cultural and social determinants strongly influence interactions between providers and patients in both pathways.

Prior successful studies in Vietnamese women have shown that while modest increases in Pap test utilization alone were achieved through promotional activity alone, when lay health worker outreach was added, far greater increases in Pap test utilization were attained. Rather than a reanalysis of the collective data, this communication considers the characteristics of care in primary studies [34,35,36,37]. This work is not a systematic review or meta-analysis, for the efficacy of the model has already been established. Rather, it is a perspective comparing selected components of the model applied to Vietnamese women and Pap smears as expanded in Figure 1, to former approaches and current advice from health system experts. Said differently, available data from prior collective experience are used to present a “pathway model” for a type of community approach to chronic disease management, with the implied hypothesis that the model may be generalizable.

## 2. Background

One health challenge that has emerged is the high prevalence of cervical cancer in Vietnamese-American women, about five-fold greater than in Caucasian women, about 43.0 versus 7.5 per 100,000 [34,35]. In California, a state with the greatest Vietnamese population, Vietnamese-American women have higher rates of invasive cervical cancer as compared to other ethnicities, demonstrating that the most undesirable consequences had in fact become reality [36]. Vietnamese-American women have unusually low rates of receiving Papanicolaou (Pap) tests as compared with other ethnicities [35,36,37]. Additional health disparities suffered by this subpopulation are tobacco use (particularly second-hand smoke from males who smoke), lung and liver cancers, hepatitis B, depression, and post-traumatic stress disorder [37]. 

The Pap test is uniformly effective as a screening test for early cervical cancer; without exception, authoritative medical bodies recommend use in women that have been sexually active and have their cervix. The American Cancer Society (ACS) recommendations for this test during the period of interest of this study included [38].

The American Cancer Society guidelines (Table 1), recommended screening with the Pap test every 1–3 years, with modification based upon individual risk factors and upon the screening history [38]. During the second period indicated in Table 1 and since 2010, the ACS screening goal was ≥97% for at least one test, and 90% for a test within the prior 3 years. However, in Vietnamese women ≥18 years in California, 2003, this rate was 70% for the prior 3 years, compared with 87% for African-American women, 85% for Hispanic women, and 84% for Caucasian women. According to Nguyen et al. the low rate of Pap tests was “associated with sociodemographic characteristics, beliefs, access, and physician characteristics” [37]. The ACS recommendations were chosen because tracked data were readily available, and were used as reference points in pertinent studies [34,35,36,37,39].

To reverse these disappointing figures, and with an emphasis on cultural issues, the questions posed are

a) What are the modifiable predisposing factors for these observations?
b) What are the barriers, facilitators, feasibilities, and interventions with their success rates?
c) Can these techniques be generalized to delivering other health messages to Vietnamese women, and possibly to other populations?

## 3. Genesis of a Community Action Plan based upon a Pathways Model

In order to answer these questions, the Vietnamese Community Health Promotion Project (VCHPP) at the University of California organized a Coalition with 10 partners, funded by the Center for Disease Control and Prevention [37]. A quasi-experimental, controlled trial was conducted from 1999–2004 to promote use of Pap tests in Vietnamese women and build community capacity. Two populations were involved: Santa Clara County (CA) Vietnamese-Americans, *n* = 102,841, and, for comparison, a matched population in Harris County (TX), *n* = 59,248. About 48% of these populations were women, and up to 50% of households were linguistically isolated. Cross-sectional telephone surveys in intervention (CA) and control (TX) communities were performed at pre- (2000) and post-intervention (2004) periods to assess intervention impact.

Prior to this, a number of factors had been identified concerning knowledge, attitudes, and beliefs in the Vietnamese culture that influenced cervical cancer screening behavior [34,35,36,37,39,4]. Many participants had not even heard of human papillomavirus (HPV), and about half of the women were unaware that HPV caused cervical cancer, or that it could be sexually transmitted. Most believed HPV was rare. Those who knew that a Pap smear could identify cervical cancer early had twice as many Pap tests as those who did not. Yet, improved education was not consistently successful in increasing Pap test rates.

Another key factor was health insurance and knowing that the Pap test was included in coverage, or financial access. A relationship with a physician was not an important determinant, but one with a provider of the desired gender and ethnicity may have been, suggesting communication patterns were significant. Reasons given by participants for not having a Pap test are summarized in Table 2. Fatalism was common theme in some series.

A Community Action Plan (CAP) was created, patterned after a “pathways model”, which was based upon two main pathways, interactions, and components (Figure 1). The medical pathway was composed of the provider and health care system, whereas the community pathway was composed of the patient and health care access. Attributes listed under each main category (for instance, “Provider” and “Patient” in Figure 1) have predisposing, enabling, and reinforcing factors. Pathway interactions occur through cultural concordance, patient education and system capacity. Of note are the positions of the patient and community; this is not a purely patient-centric model, but emphasizes the role of community in empowering the patient and facilitating patient education, understanding, and access. The entire project was considered “community-based participatory research” (CBPR) to reflect such relationships.

## 4. Components of the Program

Six components were central to the CAP in promoting Pap tests and related patient qualities: a multimedia campaign, lay health worker outreach (LHWO), a Vietnamese Pap clinic with patient navigation/registry, a Pap registry and reminder system, continuing medical education (CME) for Vietnamese physicians, and reopening a Breast and Cervical Cancer Control Clinic. The investigators relied upon their prior knowledge and experience working with this particular community to guide their choice of the components Three features of the CAP were notable: first, a recognition that simple improvements in patient/community education would not be sufficient to effect an improvement; second, that a multipronged comprehensive program that addressed all barriers identified by patients (Table 2, left column) would be necessary; and third, that the assistance of 50 trained lay health workers would be an essential asset in communicating with Vietnamese women ([37] Table 1 and Table 3, pp. 38–39) Overcoming the barriers listed in Table 2, in addition to those mentioned by Houston [39], are considered “best ways” of promoting Pap smear use.

Data were collected using previously approved guidelines in focus groups, in surveys, in LHWO feedback, and after considering suggestions from contracted agencies.

## 5. Results of the Model

Component quantitative results were significant, with modest increases in Pap tests received in women targeted through promotional activity alone, Pap tests increasing from 70.1% to 75.5%, *p* < 0.001. Far greater increases were evident in the women who were exposed to both media promotions and LHWO interventions, with Pap tests rising from 65.8% to 81.8%, *p* < 0.001. In multivariate analysis, LHWO intervention was associated with being current in the Pap test (OR, 2.68, 95% CI, 1.83–3.92). Similarly, there were many improvements seen in the cross-sectional survey results, pre- versus post-intervention (using Harris County, TX as the comparator), including awareness variables, Pap tests in the prior year, and being offered a Pap test. A most impressive qualitative result was the willingness of lay workers to continue their work unpaid, and, in some cases advancing their careers in the field, a reflection of commitment to the common cause and strong leadership. The authors considered the outcome improvements as proof of feasibility, effectiveness, and sustainability [34,35,36,37,39]. Limitations of the study included a single control community, and the unique history and distribution of the Vietnamese population as compared with other ethnicities.

In many ways, the planning and organization of this study could serve as a model for other community interventions in other targeted subpopulations with health disparities [37]. In reviewing the details, some of which are described in related publications, the authors were familiar with the community, so much so that the word immersed might be more applicable. They were diligent planners, learning from past experience, and were already armed with a potential list of barriers that needed solutions. Importantly, they followed most community intervention recommendations that have since been set forth in detail, especially accurate conceptualization of the model used, partnering, and adequate funding [40,41,42,43]. The use of lay community health workers was one of the most effective tools employed, underscoring the value of interaction in familiar language that simultaneously incorporates unique cultural “silent communication” [39]. Attention to detail was another key aspect of their intervention, even to the point of using the Vietnamese belief in “coin rubbing”, a harmless practice, to make Vietnamese women feel comfortable with their physicians [39]. Yet, the designers of the plan were comprehensive in their approach, so that they were able to effectively reach into the community to optimize results. Planning and building community capacity were important ingredients in their project. Each component of their CAP—better physician and patient knowledge, correction of misbeliefs, adjustment to ensure timely appointments, provision of transportation and family support—was considered beneficial. Their data suggest, however, that the improvement in outcomes was not due to any one single feature of their model or program, but the overall effect of the totality of participation. 

## 6. Discussion

This same team later reported results of efforts to raise Pap screening adherence in 1005 Vietnamese women randomized to either using media-based education alone or combined with LHWO [35]. The location remained Santa Clara County, and the study took place between years 2000–2004. LHW recruited 20 women from their social networks aged ≥18 years, provided informational, emotional, instrumental, and appraisal support, and met with their combined group twice over 3–4 months to promote the Pap test. Post-intervention questionnaires were used to assess exposure to media pieces, changes in awareness, knowledge, and Pap testing performance. Participants received a $60 incentive, and LHW received $1500 compensation. The media campaign was intensive, involving 15 pieces in Vietnamese over television, radio and newspapers, advertising the convenience of the CAP components mentioned in their prior publication: services, locations, and clinic details [37]. 

The investigators compared the changes in the combined intervention group with those in the media-only group using the Z test, and then used logistic regression to determine significance of impact on women becoming up-to-date on their Pap test, along with related variables. Among women who never had a Pap test at baseline, just 27.1% (*p* < **0.**001) obtained one during the study period, as compared with 46.0% in the combined group (*p* < **0.**01). However, even though the number of participants, methods, and details were different between the studies of Mock et al. [44] and the prior study of Nguyen et al. [37]. Nonetheless, the Pap testing results reported were the same: a rise of 75.1% to 75.5% (*p* < 0.001) in the media-only group as compared with a rise of 65.8% to 81.8% (*p* < 0.001) in the combined group. Similarly, significantly more women became up-to-date on their Pap test after one year in the combined group vs the media-only group, i.e., were 2.7-fold more likely to do so, a similar figure given in the earlier report. This study, however, showed that the same model could be used to address health issues other than the Pap test, in that active and passive smoking decreased significantly in both groups. In addition, among women who never had a Pap test, the LHWO effectively motivated about 50% to do so, and nearly 20% to become up-to-date, both in just 3–4 months. As mentioned, over 25% of women successfully obtained a Pap test from the media campaign alone. The synergy of both the LHWO and media campaigns were reflected in greater awareness of the viral cause of cervical cancer, use in virgins, connection with smoking, marriage, and menopausal status. 

Limitations in this study included difficulties arising from randomization when participants were from the same household, self-reporting, errors concerning whether Pap tests were actually done by physicians, the short interval between samples (3–4 months) in relation to Pap appointment delays, and variables that may have affected the results which were not included in the regression equations. In general, our observations, discussion, and perspective are also limited by the information included in the original studies.

Within the description of the Community Action plan in the large study mentioned above, heeding principles in the “Components” section, and mindful of the variables in boxes in Figure 1, it was possible to improve the rate of Pap test receipt. This was accomplished not only through (a) the 6 components of the intervention, but also by (b) addressing patient-identified barriers (Figure 1, left column), assigned to either the community pathway, medical pathway, or both. The strength of the findings is supported by the total number of participants involved, the concordance of the component studies (see below) and the robust results reported across different investigators and study designs [34,35,36,37,39,44].

One study conducted 9 focus groups with 68 participants to explore the influence of cultural context upon Vietnamese immigrant women and physician–patient communication, its significance, and salient components [39]. Importantly, these sessions were conducted in Vietnamese. The author postulated that a “silent language” consisting of “implicit, nonverbal aspects of communication”, was a stronger determinant of successful interaction between physician and patient than spoken verbal communication ([39] p.38). Three major culturally-dependent factors were identified: interaction of traditional health practices with Western medicine, predominance of polychronic time orientation (rather than completion of tasks in sequence according to a schedule), and the significant role of family members in the physician–patient relationship. Corrective measures proposed were broadening physicians’ knowledge concerning beliefs, modification of treatments to show greater cultural understanding, adjustment of clinic schedules to accommodate off-hours service, and greater inclusion of family members in decision-making discussions. These qualitative results agree with the experience and conclusions of Nguyen et al. [37] and expand upon the need for convenient scheduling of appointments. Taken together, these 3 salient contextual features and the barriers enumerated in Table 2 and Table 3 constituted a working inventory of issues to be considered in the promotion of health in this subpopulation. Similarly, the recommendations of Houston [39] added to the Community Action Plan illustrated in Figure 1 comprise a working inventory of successful actions for implementation.

Do et al. [36] conducted a population-based survey of risk factors, health beliefs, and Pap testing (*n* = 352) in Vietnamese women in Seattle during 2002. Their data confirmed the poor adherence of this population to ACS Pap guidelines [38], but also a low prevalence of knowledge concerning (a) the strong association of Pap testing with prevention, and (b) a lack of association of cervical cancer with women’s hygiene. Other traditional misbeliefs and knowledge gaps were also identified, including a failure to appreciate the unusually high incidence of cervical cancer among Vietnamese women and the viral cause of the illness. Their work reinforced the need for educational programs using appropriate communication techniques. In agreement with Houston [39] these investigators urged recognition of cultural beliefs and their incorporation into interventions to improve “silent language” as well ([36] p. 113). More insight was provided by adding “decontextualization of a health problem from the belief systems and daily routines of the target population may diminish the effectiveness of health education efforts” [36]. This observation partially accounts for the greater effectiveness of entire, comprehensive programs as opposed to naked improvements in knowledge.

More recent data confirm the principles discussed above regarding risk factors for cervical cancer in Vietnamese women, barriers (including cost), the importance of traditional beliefs, customs, knowledge, and attitudes, the need for culturally-tailored media ads, educational materials, bridging assistance for access, and local language communication and skills to customize and fully reinforce the messages [44,45,46]. The interventions in the latest iteration of the Vietnamese Women’s Health Project that took place during a one-year period from 2006 to 2007 have now been found to be cost-effective [46,47,48].

Although community-based programs are advised by major organizations and authorities, results may be variable according to the definition of end points [1]. Even when meritorious programs succeed in changing individual behavior and local policies, they may not improve health outcomes [49,50]. Further research is needed to fuller delineate application of the pathways model. 

## 7. Conclusions

These studies collectively demonstrate that full planning, organization, partnering, leadership, commitment, adequate funding, and sustainability are essential for success in eliminating health disparities. Full identification of potential barriers and methods to correct them—as determined by the population suffering from the disparities—is fundamental. A coalition of responsible stakeholders appears to be a common ingredient for improving outcomes. Since the matrix contributing to health disparities is complex and multifaceted, the solution must also target the complexities in a reciprocal fashion. In the “pathways model” the glue holding providers, community, and components together was cultural competency, patient education, and system capacity. The vehicle of improved communication to better connect with the Vietnamese people was the LHW, and the comprehensive nature of the CAP served to envelop the community as a whole. This connection was closer than in many similar applications of community involvement, and the depth and extent of the immersion of workers was similarly unusual. Logistic regressions in two of the studies illustrated the relative contributions of several of the changes made in misbeliefs, educational points, and effectiveness of the improvements made in the medical pathway. 

In this population, characterized by recognized cultural barriers contributing to low adherence and resistant to prior interventions, the pathways model may improve chronic disease outcomes. Central features are concordant with current concepts in guidelines, scientific statements, manuals, and advisories concerning the conduct of community-based research vis-à-vis social determinants of health. Given the scope and intensity of the campaigns considered, one would be inclined to anticipate similar success in eliminating health disparities with strong community involvement, perhaps with an even larger number of participants, depending upon the challenge.

With respect to the 3 research questions posed earlier, the material presented above suggests that

a) Predisposing factors contributing to the prevalence of low Pap tests initially included participant misinformation concerning the disease, testing, and effects upon outcomes, but also concerning cultural beliefs, awareness and attitudes, all of which were modifiable.
b) Individual participants identified important perceived barriers listed in Table 2. Those assigned to the community were addressed by lay health workers, educational and nonmedical staff individually. One of the most important conduits of transformation was a bond formed by the staff that spoke Vietnamese and could relate with their culture.
c) Techniques used in the pathway model can be used to deliver other health messages to Vietnamese women. However, specific data from each application, health delivery system, new population and location will be required in each instance.

## Figures and Tables

**Table 1:** Recommendations of the American Cancer Society (ACS) for Pap tests during recent periods [38]. Pap, Papanicoulou; HPV, human papillomavirus.

Period (years)	Test	Age Range(s)	Recommendation
1987–2002	Pap test	18 and over or sexually active	Yearly, but after 3 consecutive normal exams, less frequently at the discretion of the doctor
2003–2012	Pap test	Start 3 years after first vaginal intercourse but no later than 21	Yearly with conventional Pap test or every 2 years with liquid-based Pap test
		30 and over	After 3 normal results in a row, screening can be every 2 to 3 years. An alternative is a Pap test plus HPV DNA testing every 3 years.
		70 and over	After 3 normal Pap tests in a row within the past 10 years, women may choose to stop screening.

**Table 2:** Specific reasons given for not having a Pap test among Vietnamese American women [37].

Barrier	%
Feeling well	3.8
No insurance	5.5
Not suggested by physician	5.5
High cost	8.6
Lack of time	5.2
Shame/embarrassment	4.8
Lack of knowledge	3.5
No physician	3.5
Did not know where to go	2.8
Physician not speaking Vietnamese	1.4
Uncertain insurance covered Pap	1.4
No female physician	0.7
Other	2.1

**Table 3:** Eighteen major barriers identified by the Vietnamese community that were addressed in this study, Column 1, classified according to the pathway(s) used for correction, Column 2. ([37] Table 1 and Table 2, pp. 38–39).

Barrier, as Defined by Patient	Pathway Chosen for Resolution
Poor physician recommendations	Medical
Cultural incompetence by physician, e.g., insensitivity	Medical
Lack of health insurance	Medical
Cost	Medical
Poor resources for follow-up treatment	Medical
Excessive paperwork load by staff, decreasing face-to-face time	Medical
Lack of knowledge within the community	Community
Social stigma of cancer as an STD	Community
Child, elder, or sick care during appointment time	Community
Concern about the diagnosis of cancer	Community
Directions and appointment for a Pap test	Both Medical and Community
Excessive patient paperwork	Both Medical and Community
Language barrier	Both Medical and Community
Transportation need	Both Medical and Community
Desire for female physician	Both Medical and Community
Excessive appointment waiting time	Both Medical and Community
Need for different appointment time frame (unstructured)	Both Medical and Community
Modesty and other special needs	Both Medical and Community

## References

[1] Kones R., Rumana U. (2018). Cultural primer for cardiometabolic health. Health disparities, structural factors, community, and pathways to improvement. Postgrad. Med..

[2] Heiman H.J., Artiga S. (2015). Beyond Health Care: The Role of Social Determinants in Promoting Health and Health Equity.

[3] Wilkinson R., Marmot M. (2003). Social Determinants of Health: The Solid Facts.

[4] (2017). 2016 National Healthcare Quality & Disparities Report.

[5] Kilbourne A.M., Switzer G., Hyman K., Crowley-Matoka M., Fine M.J. (2006). Advancing Health Disparities Research Within the Health Care System: A Conceptual Framework. Am. J. Public Health.

[6] McGovern L., Miller G., Hugher-Cromwick P. (2014). Health Policy Brief: The Relative Contribution of Multiple Determinants to Health Outcomes. Health Aff..

[7] World Health Organization (2002). The World Health Report 2002: Reducing Risks, Promoting Healthy Life.

[8] Friel S., Marmot M.G. (2011). Action on the social determinants of health and health inequities goes global. Annu. Rev. Public Health.

[9] Davis R.S., Savannah S., Harding M., Macaysa A., Parks L. (2016). Countering the Production of Inequities: An Emerging Systems Framework to Achieve an Equitable Culture of Health.

[10] (2017). Social Determinants of Health. Healthy People 2020.

[11] (2017). Program Planning. Health People 2020.

[12] Ahnquist J., Wamalal S., Lindstrom M. (2012). Social determinants of health—A question of social or economic capital? Interaction effects of socioeconomic factors on health outcomes. Soc. Sci. Med..

[13] Robert Wood Johnson Foundation (RWJF) (2017). How We Work Building a Culture of Health.

[14] Chang W.C., Fraser J.H. (2017). Cooperate! A paradigm shift for health equity. Int. J. Equity Health.

[15] Institute of Medicine (2003). The Future of the Public’s Health in the 21st Century.

[16] Schroeder S.A. (2007). Shattuck Lecture. We can do better—Improving the health of the American people. N. Engl. J. Med..

[17] Braveman P., Gottlieb L. (2014). The Social Determinants of Health: It’s Time to Consider the Causes of the Causes. Public Health Rep..

[18] World Health Organization (2010). A Conceptual Framework for Action on the Social Determinants of Health.

[19] Thomas S.B., Quinn S.C., Butler J., Fryer C.S., Garza M.A. (2011). Toward a fourth generation of disparities research to achieve health equity. Annu. Rev. Public Health.

[20] Wilson L. (2013). Finding Answers: Disparities Research for Change Progress Report.

[21] Cockerham W.C., Hamby B.W., Oates G.R. (2017). The Social Determinants of Chronic Disease. Am. J. Prev. Med..

[22] Taylor L.A., Tan A.X., Coyle C.E., Ndumele C., Rogan E., Canavan M., Curry L.A., Bradley E.H. (2016). Leveraging the Social Determinants of Health: What Works?. PLoS ONE.

[23] Scribner R.A., Simonsen N.R., Leonardi C. (2017). The Social Determinants of Health Core: Taking a Place-Based Approach. Am. J. Prev. Med..

[24] (2017). Healthy People 2020. Access to Health Services.

[25] Hassan A., Scherer E.A., Pikcilingis A., Krull E., McNickles L., Marmon G., Woods E.R., Fleegler E.W. (2015). Improving Social Determinants of Health: Effectiveness of a Web-Based Intervention. Am. J. Prev. Med..

[26] Smith M.D., Institute of Medicine (US) (2013). Committee on the Learning Health Care System in America. Best Care at Lower Cost: The Path to Continuously Learning Health Care in America.

[27] Ayanian J.Z., Markel H. (2016). Donabedian’s Lasting Framework for Health Care Quality. N. Engl. J. Med..

[28] Robert Wood Johnson Foundation, Johns Hopkins Bloomberg School of Public Health (2010). Chronic Care: Making the Case for Ongoing Care. https://www.rwjf.org/content/dam/farm/reports/reports/2010/rwjf54583.

[29] Knapper J.T., Ghasemzadeh N., Khayata M., Patel S., Quyyumi A., Mendis S., Mensah G.A., Taubert K., Sperling L. (2015). Time to Change Our Focus. Defining, Promoting, and Impacting Cardiovascular Population Health. J. Am. Coll. Cardiol..

[30] Kones R., Rumana U. (2017). Cardiometabolic diseases of civilization: History and maturation of an evolving global threat. An update and call for action. Ann. Med..

[31] (2014). Center for Advanced Research on Language Acquisition.

[32] Verma A., Griffin A., Dacre J., Elder A. (2016). Exploring cultural and linguistic influences on clinical communication skills: A qualitative study of International Medical Graduates. BMC Med. Educ..

[33] Pew Research Center (2013). Vietnamese Americans. http://www.pewsocialtrends.org/asianamericans-graphics/vietnamese/.

[34] Mock J., McPhee S.J., Nguyen T., Wong C., Doan H., Lai K.Q., Nguyen K.H., Nguyen T.T., Bui-Tong N. (2007). Effective lay health worker outreach and media-based education for promoting cervical cancer screening among Vietnamese American women. Am. J. Public Health.

[35] Taylor V.M., Schwartz S.M., Yasui Y., Burke N., Shu J., Lam D.H., Jackson J.C. (2004). Pap testing among Vietnamese women: Health care system and physician factors. J. Community Health.

[36] Do H.H., Taylor V.M., Burke N., Yutaka Y., Schwartz S.M., Jackson J.C. (2007). Knowledge about cervical cancer risk factors, traditional health beliefs, and Pap testing among Vietnamese American women. J. Immigr. Minor. Health.

[37] Nguyen T.T., McPhee S.J., Bui-Tong N., Luong T.N., Ha-Iaconis T., Nguyen T., Wong C., Lai K.Q., Lam H. (2006). Community-based participatory research increases cervical cancer screening among Vietnamese-Americans. J. Health Care Poor Underserved.

[38] American Cancer Society (ACS) History of ACS Recommendations for the Early Detection of Cancer in People Without Symptoms. https://www.cancer.org/healthy/find-cancer-early/cancer-screening-guidelines/chronological-history-of-acs-recommendations.html.

[39] Houston H.R. (2002). Health care and the silent language of Vietnamese immigrant consumers. Bus. Prof. Commun. Q..

[40] Baciu A., Negussie Y., Geller A., Weinstein J.N. (2017). Communities in Action: Pathways to Health Equity.

[41] Centers for Disease Control and Prevention (CDC). CDC Community Health Improvement Navigator, 2015. http://www.cdc.gov/chinav/.

[42] Centers for Disease Control and Prevention (2017). The Community Guide. What Works to Promote Health. https://www.thecommunityguide.org/.

[43] Community Tool Box (2016). Chapter 1. Section 7. Working Together for Healthier Communities: A Framework for Collaboration among Community Partnership, Support Organizations, and Funders.

[44] Do M. (2015). Predictors of cervical cancer screening among Vietnamese American women. J. Immigr. Minor. Health.

[45] Ma G.X., Fang C.Y., Feng Z., Tan Y., Gao W., Ge S., Nguyen C. (2012). Correlates of Cervical Cancer Screening among Vietnamese-American Women. Infect. Dis. Obstet. Gynecol..

[46] Taylor V.M., Jackson J.C., Yasui Y., Nguyen T.T., Woodall E., Acorda E., Li L., Ramsey S. (2010). Evaluation of a cervical cancer control intervention using lay health workers for Vietnamese American women. Am. J. Public Health.

[47] National Cancer Institute (2013). Research-tested Intervention Programs. Vietnamese Women’s Health Project. https://rtips.cancer.gov/rtips/programDetails.do?programId=1427816.

[48] Scoggins J.F., Ramsey S.D., Jackson J.C., Taylor V.M. (2010). Cost effectiveness of a program to promote screening for cervical cancer in the Vietnamese-American population. Asian Pac. J. Cancer Prev..

[49] Fr C.E., Nikpay S.S., Leslie E., Buntin M.B. (2018). Evaluating Community-Based Health Improvement Programs. Health Aff..

[50] Kones R., Rumana U., Merino J. (2014). Exclusion of ‘nonRCT evidence’ in guidelines for chronic diseases—Is it always appropriate? The Look AHEAD study. Curr. Med. Res. Opin..

